# IIMLP: integrated information-entropy-based method for LncRNA prediction

**DOI:** 10.1186/s12859-020-03884-w

**Published:** 2021-05-13

**Authors:** Junyi Li, Huinian Li, Xiao Ye, Li Zhang, Qingzhe Xu, Yuan Ping, Xiaozhu Jing, Wei Jiang, Qing Liao, Bo Liu, Yadong Wang

**Affiliations:** 1 School of Computer Science and Technology, Harbin Institute of Technology (Shenzhen), Shenzhen, 518055 Guangdong China; 2 Center for Bioinformatics, School of Computer Science and Technology, Harbin Institute of Technology, Harbin, 150001 Heilongjiang China

**Keywords:** Long non-coding RNA, Information entropy, Generalized topological entropy, Machine learning

## Abstract

**Background:**

The prediction of long non-coding RNA (lncRNA) has attracted great attention from researchers, as more and more evidence indicate that various complex human diseases are closely related to lncRNAs. In the era of bio-med big data, in addition to the prediction of lncRNAs by biological experimental methods, many computational methods based on machine learning have been proposed to make better use of the sequence resources of lncRNAs.

**Results:**

We developed the lncRNA prediction method by integrating information-entropy-based features and machine learning algorithms. We calculate generalized topological entropy and generate 6 novel features for lncRNA sequences. By employing these 6 features and other features such as open reading frame, we apply supporting vector machine, XGBoost and random forest algorithms to distinguish human lncRNAs. We compare our method with the one which has more K-mer features and results show that our method has higher area under the curve up to 99.7905%.

**Conclusions:**

We develop an accurate and efficient method which has novel information entropy features to analyze and classify lncRNAs. Our method is also extendable for research on the other functional elements in DNA sequences.

## Background

According to the central dogma of molecular biology, genetic information is stored in protein-coding genes [1]. Therefore, non-coding RNAs were considered to be transcriptional noises for a long time. In the past decade, this traditional view has been challenged [2]. There is increasing evidence shows that non-coding RNAs play a key role in a variety of basic and important biological processes [3]. Moreover, the proportion of non-protein coding sequences increases with the complexity of the organism [4]. Non-coding RNAs can be further divided into short non-coding RNAs and long non-coding RNAs (lncRNAs) based on whether the length of the transcript exceeds more than 200 nucleotides (nt).

Recently, long non-coding RNA has attracted great attention from researchers, as more and more research results indicate that mutations and dysregulation of these long non-coding RNAs are associated with the development of various complex human diseases such as cancers, Alzheimer's disease and cardiovascular diseases [5]. Therefore, accurate prediction of lncRNAs is very important in lncRNA studies [6–8].

Various lncRNA prediction methods have been proposed by using experimental techniques and biological data. For example, the discovery of two well-known lncRNAs, H19 and X-inactive specific transcripts can be traced back to traditional genetic mapping in the early 1990s [9]. Guttman et al. developed a functional genomics approach that assigns putative functions to each large intervening lncRNA [10]. Cabili et al. proposed a comprehensive approach to construct large non-coding RNA catalogs of human intercropping, which includes more than 8000 large intervening lengths in 24 different human cell types and tissues [11].

However, the method of biological experiment is costly, time-consuming and laborious, which is not conducive to large-scale application. In the era of bio-big data, in order to make better use of the existing sequence resources of lncRNA, many computational methods based on machine learning have been proposed by researchers.

In 2013, CPAT was implemented by L. Wang et al., which is a potential evaluation tool for protein coding and includes the feature of Open Reading Frame (ORF) [12]. In molecular biology, ORF starts from the start codon and ends at a stop codon, is a basic sequence in the DNA sequence which has protein coding potential. The classification model of CPAT takes the Supporting Vector Machine (SVM) basis function with standard radial as kernel. In 2014, PLEK was implemented by A. M. Li et al., which analyzes transcripts by using k-mer scheme and sliding window [13]. The classification model of PLEK is an SVM with a radial kernel function.

In 2015, LncRNA-ID was implemented by Achawanantakun et al. [14]. LncRNA-ID can be classified according to ORF, ribosome interaction and protein conservation. The use of Random forests improves the classification model of LncRNA-ID, which helps LncRNA-ID to efficiently process unbalanced training data.

In 2017, Hugo W. Schneider et al. proposed an SVM-based method for the prediction of lncRNAs [15]. It uses the k-mer protocol and features derived from the ORF to analyze the transcript. These features are divided into two groups. The first set derives from the four characteristics of the ORF: the first ORF length; the relative length of the first ORF; the longest ORF length; the longest ORF relative length. The second group is based on the k-mer feature extraction scheme, where k = 2, 3, 4, a total of 336 nucleotide patterns of different frequencies: 16 dinucleotide pattern frequencies; 64 trinucleotide mode frequencies; and 256 four nucleotide mode frequencies. The first ORF relative length and the frequency of the nucleotide pattern selected by PCA were used as features for these two sets of features.

In our study, we use a lncRNA prediction method by integrating information-entropy-based features and machine learning algorithms. We calculate generalized topological entropy and generate 6 novel features for lncRNA sequences. By employing these 6 features and other features such as ORF, we apply SVM, XGBoost and Random Forest algorithms to distinguish human lncRNAs. We compare our method with the one which has more k-mer-based features. Results show that our method has higher Area Under the Curve (AUC) score up to 99.7905%. Our accurate and efficient method which has novel information entropy features and is extendable for research on the other functional elements in DNA sequences.

## Results

### Feature selection by XGBoost

**Fig. 1:**
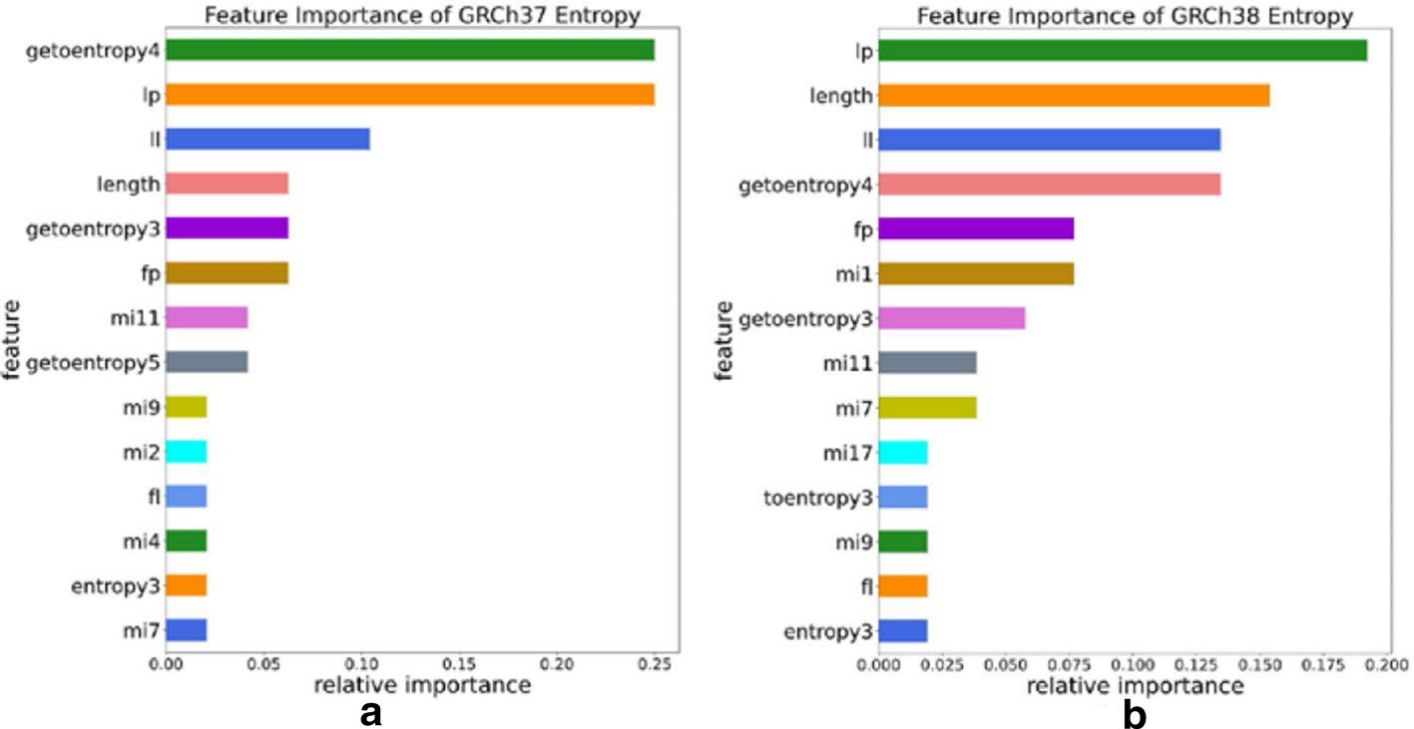
**a** Feature importance of human GRCh37 data based on information entropy and ORF; **b** feature importance of human GRCh38 data based on information entropy and ORF

Random forest and XGBoost both belong to decision tree algorithms. The decision tree algorithm calculates the information entropy gain that can be obtained by dividing a certain feature before each split, and automatically selects the feature that can maximize the information entropy gain for division. During this process, we can also calculate the importance of each feature according to the division. We used the XGBoost built-in function in Python to visualize the importance ranking for each feature. The results show that compared to the traditional features, the feature based on information entropy we proposed shows more important in the classification task. The feature importance ranking is shown in Fig. 1.

Figure 1 shows that the first four importance features are: length, fourth of generalized topological entropy, and longest ORF Relative length (lp), the length of the longest ORF (ll). And the two versions of human data have a certain degree of consistency in the selection of features. In the K-mer comparison experiment we designed, we use the same method for feature selection and the selected feature importance are listed in the additional files.

### Machine learning model training results comparison

We apply SVM, XGBoost and Random Forest algorithms with 35 features to distinguish human lncRNAs for GRCh37 version and compare with the ones with K-mer features.

**Fig. 2:**
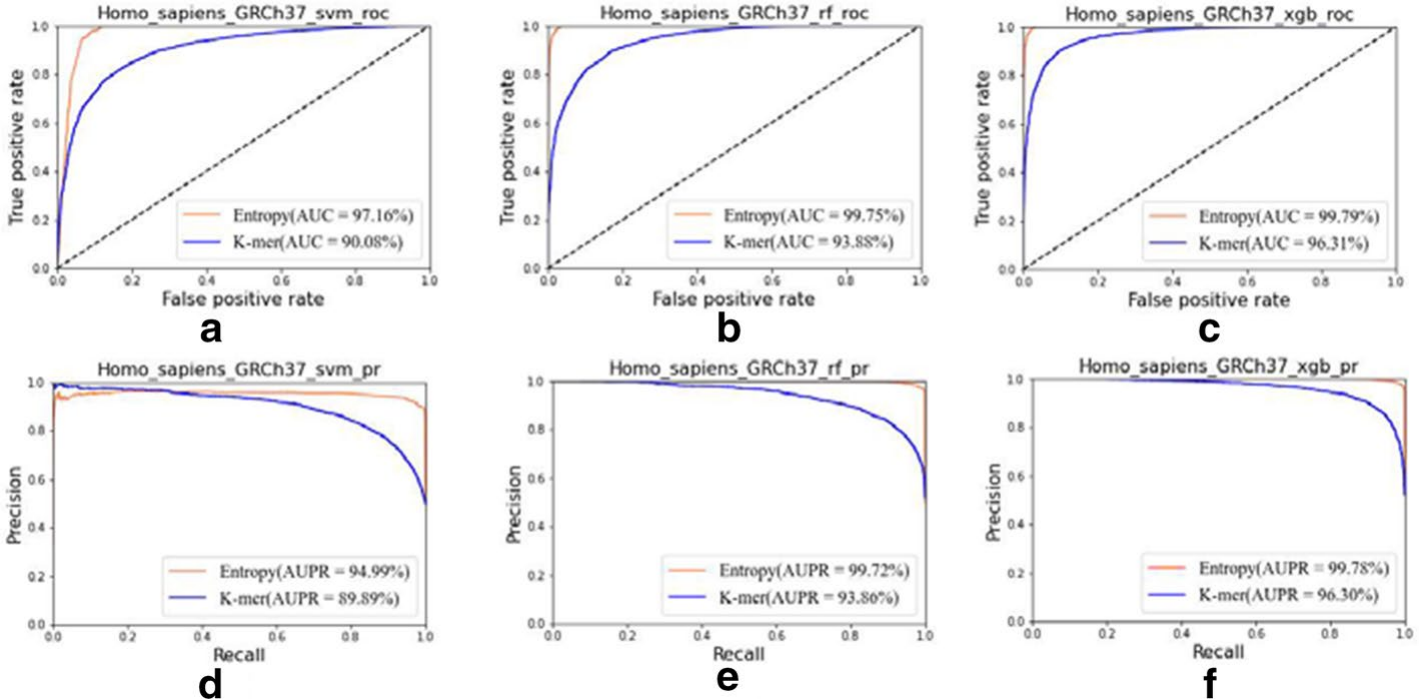
Experimental results based on GRCh37 version of human species: **a** ROC curve of svm algorithm; **b** ROC curve of random forest algorithm; **c** ROC curve of XGBoost algorithm; **d** PR curve of svm algorithm; **e** PR curve of random forest algorithm; **f** PR curve of XGBoost algorithm

It can be seen from Fig. 2 that the method based on the combination of information entropy and ORF extracts features is superior to the method based on K-mer extraction features in general, which are described as follows: (1) In Fig. 2a–c, the AUC value of the information entropy is up to 99.7905%, and the AUC value of K-mer is 96.3130% at most; (2) For the same training algorithm, the AUC value of the information entropy is larger than the AUC value of K-mer one. The maximum difference is 7.0820% and the average difference is 5.4766%; (3) In Fig. 2d–f, the AUPR value of the information entropy is up to 99.7792%, and the AUPR value of K-mer is 96.3035% at most; (4) In Fig. 2d–f, the AUPR value of information entropy is larger than the AUPR value of K-mer one, with a maximum difference of 5.8724% and an average difference of 4.8184%.

We also apply SVM, XGBoost and Random Forest algorithms with 35 features to distinguish human lncRNAs for GRCh38 version and do the similar comparison with the ones with K-mer features.

**Fig. 3:**
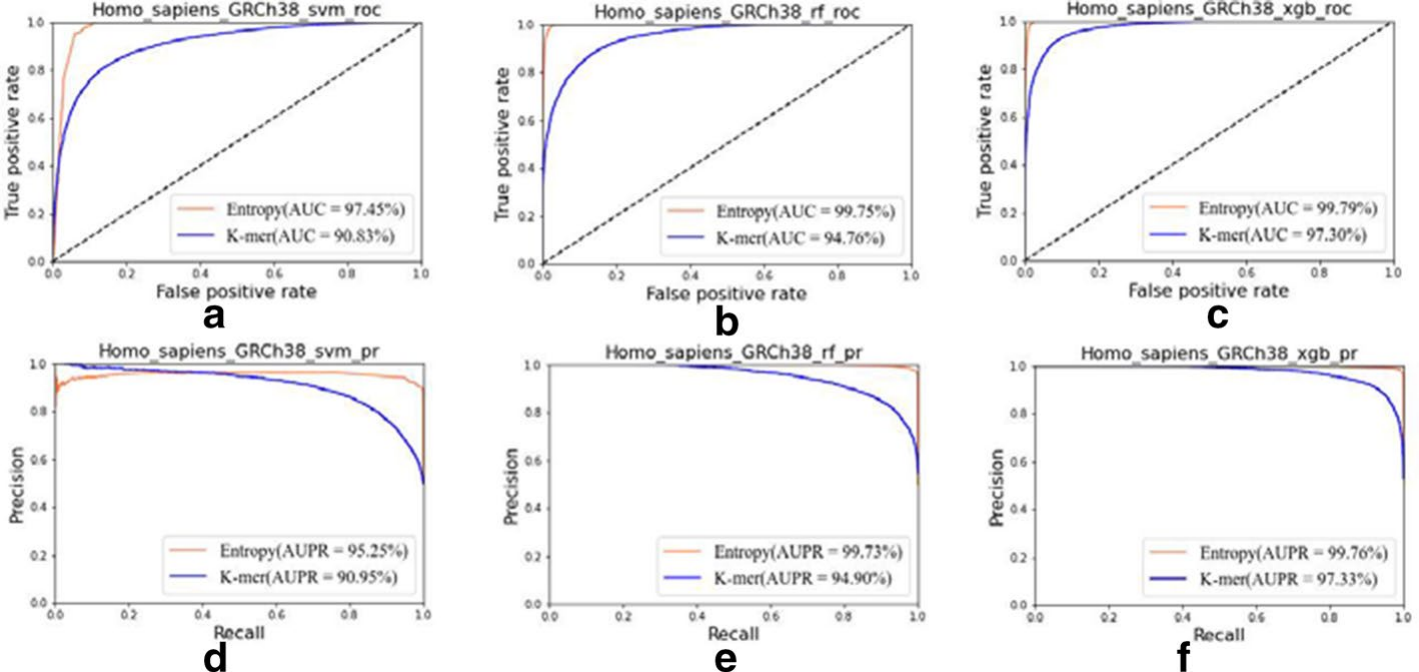
Experimental results based on GRCh38 version of human species: **a** ROC curve of svm algorithm; **b** ROC curve of random forest algorithm; **c** ROC curve of XGBoost algorithm; **d** PR curve of svm algorithm; **e** PR curve of random forest algorithm; **f** PR curve of XGBoost algorithm

We use ROC index as an evaluation indicator, because the ROC curve can easily detect the influence of any threshold on the generalization performance of the learner, which helps to select the best threshold. The closer the ROC curve is to the upper left corner, the higher the accuracy of the model. By comparing the ROC curves of different learners, the pros and cons of different learners can be visually identified. Figure 3 shows that for the GRCh38 version of the human species, the method based on the combination of information entropy and ORF is better to the method based on K-mer extraction features too: (1) In Fig. 3a–c, the AUC value of the information entropy is 99.7887% at the maximum, and the AUC value of K-mer is 97.3003% at the maximum; (2) In Fig. 3a–c, the AUC value of the information entropy method is larger than the AUC value of K-mer one as the maximum difference is 6.6198% and the average difference is 4.6982%; (3) In Fig. 3d–f, the AUPR value of the information entropy is up to 99.7606%, and the AUPR value of K-mer is 97.3299% at most; (4) In the Fig. 3d–f, the AUPR value of information entropy is larger than the AUPR value of K-mer one, with a maximum difference of 4.8293% and an average difference of 3.8553%.

**Fig. 4:**
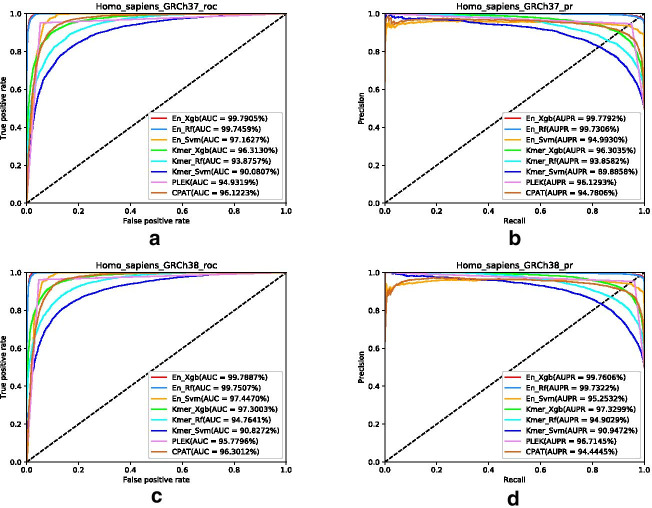
**a** ROC curve of GRCh37; **b** PR curve of GRCh37; **c** ROC curve of GRCh38; **d** PR curve of GRCh38

**Fig. 5:**
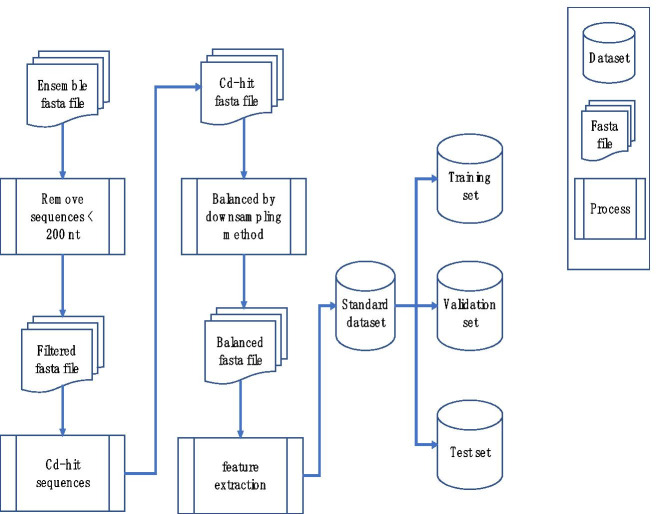
Data processing flow chart

To further investigate our integrated features and method, we apply available methods PLEK and CPAT for comparison. The results are shown in Fig. 4. It demonstrates that XgBoost for integrated features has the best AUC and PR values. In Fig. 4a, b, the AUC value of PLEK is 1.0562% greater than that of K-mer RF, while it is 1.1904% less than that of CPAT. The PR value of PLEK is 1.3487% greater than that of CPAT. In Fig. 4c, d, the AUC value of PLEK is 1.0155% greater than that of K-mer RF, while it is 0.5216% less than that of CPAT. The PR value of PLEK is 2.27% greater than that of CPAT. It is worth noting that the running time of PLEK on these 35 features is 9 days and the other methods are much less time-consuming.

## Conclusions

In this paper, an effective lncRNA predictor IIMLP is proposed. In order to obtain more accurate and realistic prediction results, we use the CD_HIT tool to perform de-redundancy operations on nucleic acid sequences. Characteristics features are extracted from the nucleic acid sequence itself, and the topological entropy and generalized topological entropy are regarded as new information theoretical features. We combine 35 features to train the classifier. Feature selection and classifier training are performed using SVM, random forest and XGBoost machine learning methods. Compared with the K-mer control experiment, we use 49 fewer features and speed up the training process. One advantage of our approach is that we only use features that are calculated directly from the sequence itself. Our method not only achieves good performance in lncRNA prediction, but also is extendable for research on other functional elements in DNA sequences.

## Materials and methods

### Data sets

We use the dataset from the Ensembl [16] database for model training: human (Homo sapiens) assemblies GRCh37 (release-75) and GRCh38 (release-91). These categorical FASTA files of transcripts contain lncRNAs and protein-encoding transcripts (PCTs) (shown in Table 1). In this project, we consider lncRNAs as positive samples and PCTs as negative samples.

**Table 1:** Categorical original FASTA files of transcripts

Transcripts types	GRCh37 ncRNAs	GRCh37 PCTs	GRCh38 ncRNAs	GRCh38 PCTs
Number	34917	104763	37297	104817

### Data processing with CD-HIT

CD-HIT is a widely used program for clustering biological sequences to reduce sequence redundancy and improve the performance of other sequence analyses. CD-HIT was originally developed to clustering protein sequences to create a reduced reference database [17, 18] and then extended to support clustering nucleotide sequences and compare two data sets [19].

Currently, the CD-HIT package has many programs: cd-hit, cd-hit-2d, cd-hit-est, cd-hit-est-2d, cd-hit-para and so on. In this project, we use cd-hit-est to clustering nucleic acid sequences. The purpose is to perform de-redundancy operations on nucleic acid sequences to ensure the accuracy of the model for machine learning training.

Data processing is briefly described as shown in Fig. 5.

Firstly, we remove all sequences shorter than 200 nt from the original files (as shown in Table 1). Secondly, we use the “cd-hit-est” program in the CD-HIT package to perform deduplication operations, the purpose of this step is to prevent the model from overfitting a certain part of the sample. Thirdly, we randomly down sampled the larger number of the two types of sample to keep the number of samples of lncRNAs and PCTs consistent. This step is very important, because unbalanced samples may cause our classification model to be very biased and it cannot to be seen from some commonly used indicators. For example, in a positive-to-negative sample with ratio of 99:1, as long as all samples are judged to be positive the accuracy of classification can reach to 99%, this is obviously not caused by our use of better features or better models. In the fourth step, feature extraction is performed to obtain training data. Table 2 shows the changes in the number of nucleic acid sequences in the FASTA file after data processing.

**Table 2:** Categorical FASTA files of transcripts after data processing

Transcripts types	GRCh37 ncRNAs	GRCh37 PCTs	GRCh38 ncRNAs	GRCh38 PCTs
After removing short	24,513	94,830	28,628	94,527
After deduplication	21,965	41,134	24,863	41,200
After data balancing	21,965	21,965	24,863	24,863

### Novel features extracted from modified topological entropy and modified generalized topological entropy

Koslicki defined topological entropy of a sequence as follows [20]:

1 $$H_{top\left( w \right)} = \frac{{\log_{4} \left( {p_{{w_{1}^{{4^{n} + n - 1}} }} \left( n \right)} \right)}}{n}$$

The length of a finite sequence is $\upomega$ and the length of a sub sequence is n. $\upomega$ satisfies that $4^{{n_{\omega } }} + n_{\omega } - 1 \le \left| {\upomega } \right| \le 4^{{n_{\omega } + 1}} + \left( {n_{\omega } + 1} \right) - 1$. $p_{{w_{1}^{{4^{n} + n - 1}} }} (n)$ is the number of sub sequences with length n which are in the first $4^{{{\text{n}}_{{\upomega }} }} + {\text{n}}_{{\upomega }} - 1$ bp of ω. In our project, we choose the length of sub sequence *n* = 3, 4, 5 to calculate three novel features.

Our previous work shows generalized topological entropy is a complete form of topological entropy [21, 22] and it is defined as:

2 $$H_{{n_{\omega } }}^{\left( k \right)} \left( \omega \right) = \frac{1}{k}\mathop \sum \limits_{{i = n_{\omega } - k + 1}}^{{n_{\omega } }} \frac{{\log_{4} (p_{\omega } \left( i \right))}}{i}$$

In Eq. 2, n_*ω*_ fulfils $4^{{{\text{n}}_{{\upomega }} }} + {\text{n}}_{{\upomega }} - 1 \le \left| {\upomega } \right| \le 4^{{{\text{n}}_{{\upomega }} + 1}} + \left( {{\text{n}}_{{\upomega }} + 1} \right) - 1$ and *k* ≤ n_*ω*_. And p_*ω*_(i) is the number of sub sequences of length i within first $4^{{{\text{n}}_{{\upomega }} }} + {\text{n}}_{{\upomega }} - 1$ bp of ω.

We modified both topological entropy and generalized topological entropy to emphasize repetition subsequences. In our calculation we ignored subsequences with lower appearance frequencies. That means, this kind of sub sequences will not be included in the entropy calculation if the frequency of a subsequence is smaller than $4^{{{\text{n}}_{{\upomega }} }} /{\upomega }$. From Eq. (2), *k* = 3, 4, 5 was chosen and 3 novel features based on modified generalized topological entropy are calculated.

### Combination of information theoretic features

It is very difficult to perform lncRNA prediction based only on the 6 previously-extracted features. The best approach is to combine them with other commonly used informational theory features and ORF-related features of lncRNAs to obtain better performance classifiers. Common features based on information theory and entropy have been proposed in computational biology and bioinformatics to analyze and measure structural properties in the transcripts. Different complexity calculations reveal different aspects of transcript specificity. In our project, we also employ useful theoretical information features used by Henkel et al. [23]. The features used in this article constitute a 35-dimensional vector, which includes: 1 sequence length feature, 4 ORF features [15], 4 Shannon entropy (SE) features [24], 3 topological entropy (TE) features [20], 3 generalized topology Entropy (GTE) features [21], 17 mutual information (MI) features [25] and 3 Kullback–Leibler divergence (KLD) features [26]. In order to better illustrate the superiority of our research, the K-mer feature was chosen as a comparative test. In the comparative experiment, there are a total of 84 nucleotide patterns with different frequencies when k is 1, 2 and 3. They are 4 single nucleotide pattern frequency, 16 dinucleotide pattern frequencies and 64 trinucleotide pattern frequencies. Calculation of all features are listed in the additional files.

### Support vector machine, random forest and XGBoost algorithms for classification procedure

**Fig. 6:**
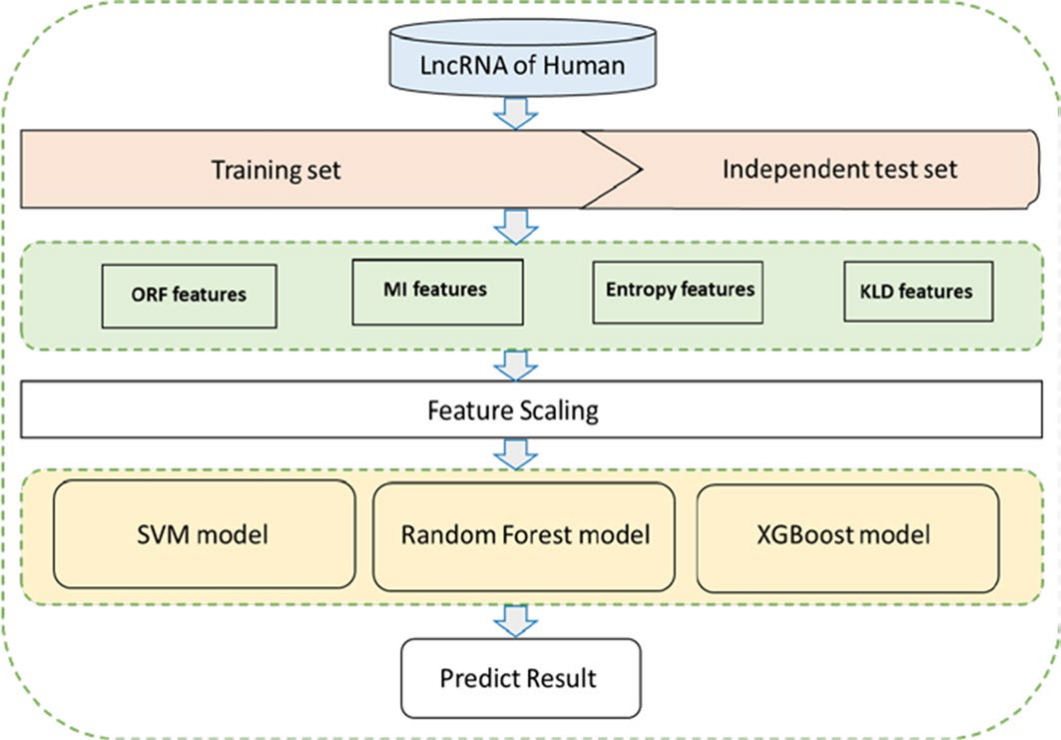
The flowchart of human LncRNA prediction based on combination of information entropy and ORF features

SVM [27], Random Forest [28] and XGBoost [29] are widely used machine learning algorithms, which we use to identify lncRNAs and PCTs. The SVM algorithm is a supervised learning model related to the relevant learning algorithm, which can analyze data, identify patterns, and use for classification and regression analysis. The RF algorithm is an integrated learning method for classification tasks. It constructs a large number of decision trees when training data, and outputs the classes of each tree. The XGBoost algorithm predicts output variables based on various rules organized in a tree structure. Moreover, XGBoost's learning method does not require linear features or linear interaction between features. It is a gradient enhancement which can accelerate tree construction and propose a new tree search distributed algorithm. In our work, all samples are described by the 35 features. And the entire process used in our study is shown in Fig. 6.

### Machine learning parameter adjustment

K-fold cross validation is used to adjust parameters. The initial sample is segmented into K subsamples, a single subsample is retained as data for the validation model, and the other K − 1 samples are used for training. Cross-validation is repeated K times, each sub-sample is verified once, and the average K-time results are used or other combinations are used. Finally get a single estimate. The advantage of this method can be repeated using randomly generated subsamples for training and verification. Verify the results each time. In order to ensure the accuracy of the model, this paper uses three trainers to build the classification model. The cross-validation used during the experiment was a fivefold cross-validation.

In order to better select the appropriate machine learning model parameters, this article uses the GridSearch method. For SVM, there are only two parameters that need to be adjusted. This article uses GridSearch directly here. For XGBoost and Randomforest, there are too many parameters to adjust, and we use some GridSearch in order to get the appropriate parameters better and faster. The central idea is to perform GridSearch on some parameters. At first, a part or the parameters were fixed and we adjust a parameter to optimize the performance of the classifier. In this process, we use five-fold cross-validation to evaluate the new capabilities of the model. Then we perform the same operations as the previous parameters until all parameters are optimal.

## Data Availability

All datasets and additional files are available at: https://github.com/lihuinian/lncRNAIdentification.

## Abbreviations

lncRNA: Long non-coding RNA
TE: Topological entropy
SVM: Supporting vector machine
RF: Random forest
AUC: Area under the curve
AUROC: Area under the receiver operating characteristic curve
RF: Random forest
